# Competitive Swarm Optimizer Based Gateway Deployment Algorithm in Cyber-Physical Systems

**DOI:** 10.3390/s17010209

**Published:** 2017-01-22

**Authors:** Shuqiang Huang, Ming Tao

**Affiliations:** 1 Department of Optoelectronic Engineering, Jinan University, Guangzhou 510632, China; 2 College of Computer and Network Security, Dongguan University of Technology, Dongguan 523808, China; ming.tao@mail.scut.edu.cn

**Keywords:** cyber-physical system, particle swarm optimization (PSO), competitive swarm optimizer (CSO), gateway deployment, geometric *K*-center, covering radius

## Abstract

Wireless sensor network topology optimization is a highly important issue, and topology control through node selection can improve the efficiency of data forwarding, while saving energy and prolonging lifetime of the network. To address the problem of connecting a wireless sensor network to the Internet in cyber-physical systems, here we propose a geometric gateway deployment based on a competitive swarm optimizer algorithm. The particle swarm optimization (PSO) algorithm has a continuous search feature in the solution space, which makes it suitable for finding the geometric center of gateway deployment; however, its search mechanism is limited to the individual optimum (pbest) and the population optimum (gbest); thus, it easily falls into local optima. In order to improve the particle search mechanism and enhance the search efficiency of the algorithm, we introduce a new competitive swarm optimizer (CSO) algorithm. The CSO search algorithm is based on an inter-particle competition mechanism and can effectively avoid trapping of the population falling into a local optimum. With the improvement of an adaptive opposition-based search and its ability to dynamically parameter adjustments, this algorithm can maintain the diversity of the entire swarm to solve geometric *K*-center gateway deployment problems. The simulation results show that this CSO algorithm has a good global explorative ability as well as convergence speed and can improve the network quality of service (QoS) level of cyber-physical systems by obtaining a minimum network coverage radius. We also find that the CSO algorithm is more stable, robust and effective in solving the problem of geometric gateway deployment as compared to the PSO or Kmedoids algorithms.

## 1. Introduction

Cyber-Physical Systems (CPSs) have great influence on the way we observe and change the world. Deploying cyber-physical systems (CPS) have emerged as a new trend in the development of industrial informatization and has attracted the attention of both academia and industry [1,2]. In a CPS, information regarding the monitoring area within a local area is acquired using a large number of micro-sensor nodes and a multi-hop, self-organizing wireless sensor network is created through wireless communication, and transport delay, data security are important topics [3,4,5]. When evaluating CPS performance, the gateway is typically the bottleneck because the majority of the network traffic converges at this point. A non-uniform service quality exists among the notes, in which the nodes that are closer to the gateway receive better service, while the nodes that are farther away receive lower-quality service. Thus, the deployment of the gateway has a great impact on the real-time performance and reliability of CPS information transmission.

In CPS, data transmission usually takes advantage of wireless communication. Due to the rapid development of wireless communication technology, CPS with multiwireless technology or heterogeneous sensors coexisting become familiar. Different wireless communication technologies are complementary but also bring challenges. One of the biggest challenges is that these technologies cannot communicate mutually. Under this condition, a wireless mesh network (WMN) is a feasible wireless network architecture. The WMN is a new type of wireless access technology that can effectively connect various types of sensing devices and has become widely used in CPS. Its advantages include the multi-hop feature, easy maintenance, wide coverage and low deployment cost. The majority of the traffic in a WMN converges at the gateway; therefore, the nodes that are closer to the gateway receive a better service quality, whereas the service quality for the nodes that are further away from the gateway is lower. Thus, the gateway remains the bottleneck, restricting the network performance. This highlights the importance of gateway deployment in WMN design. Currently, there are two types of WMN gateway deployments [6]: one is to select *K* network nodes from the existing network nodes as the gateways, referred to as the vertex *K*-center problem, and the method is searching for local optimal in a discrete space. The other is to choose *K* positions from the plane in which the node is located to deploy gateway nodes, referred to as the geometric *K*-center problem. This method is searching for a global optimal in a continuous space. The former is simple and easy to implement but has poor performance in comparison to the latter. While the latter may perform better, it is difficult to solve.

Many developments have resulted from research on vertex *K*-center WMN gateway deployment. Node clustering, in which network nodes are divided into different clusters and the center of the cluster becomes the gateway location, was proposed by Bejerano and has been widely applied in various studies [7]. He et al. [8] proposed degree-based and weight-based heuristic algorithms, using the network adjacency matrix as the data model in order to minimize the number of gateways and to minimize the longest path between the nodes and the gateway. A zero-degree gateway selection method was also proposed [9], in which network nodes were grouped according to their connectivity, and then a gateway was selected for each group. An integer programming model for gateway selection was proposed by Valerio [10]. The gateway deployment problem was also studied in combination with the routing problem [11], and the shortest path cost matrix was used to solve the problem. Some intelligent algorithms (such as simulated annealing (SA), PSO) are used to solve the vertex *K*-center problem [12,13].

In the vertex *K*-center problem, gateway nodes are selected from existing nodes, so their optimization is constrained by the structure of the network. Thus, a globally optimal solution is typically not found. It has been proposed that appropriate locations in the geometric plane of a WMN should be selected to deploy the gateway, and the gateway deployment problem based on this method is known as the geometric *K*-center problem. Gateway deployment method based on the geometric *K*-center problem has been shown to be more adaptable, as well as more complex, than the ones based on the vertex *K*-center problem because it is searching for global optimal in continuous space. The geometric *K*-center problem of wireless network gateway deployment is essentially a classical mathematical problem. The basic properties and the computational complexity of the geometric *K*-center problem using graph theory and using the unit disc theory to solve the geometric *K*-center problem have been researched by Plesnik and Megiddo [14,15,16]. The concept of a maximum common coverage area was proposed using the unit disc theory, and the relevant theoretical analysis is provided in reference [6]. Some methods aiming to solve single-hop wireless network gateway deployment are proposed [17,18]. The gateway deployment methods described above are applicable to general wireless networks. However, they are not suitable for multihopWMN. In order to convert the geometric *K*-center problem into a vortex *K*-center problem, a planar cutting method was proposed using the unit disc theory [19], and the geometric *K*-center problem was solved directly using the spatial continuous search ability of PSO [20].

The PSO algorithm is a classic intelligent optimization algorithm. Its implementation is simple and easy, as it does not require gradient information and only requires a few parameters. The characteristics of real number coding are suitable for solving continuous optimization and other problems. However, PSO is prone to trapping in a local optimum and many strategies have been proposed in order to improve this, including addressing the inertia weight, an asynchronous time-varying learning factor, and the development of a PSO with a contraction learning factor and domain topology [21,22]. These improvements shown, however, have not been particularly satisfactory. In the study described in reference [23], the individual optimum pbest and population optimum gbest in the PSO algorithm were eliminated, and a competition mechanism was introduced into the particle swarm, which essentially changed the particle search mechanism: in short, a competition-based CSO (competitive swarm optimizer) algorithm was proposed in an effort to effectively avoid precocious particles and local optimum trapping, as well as to lower the computation cost. This paper adopts a novel CSO algorithm in order to solve the problem of WMN gateway deployment.

## 2. CPS WMN Gateway Deployment Model

**Figure 1:**
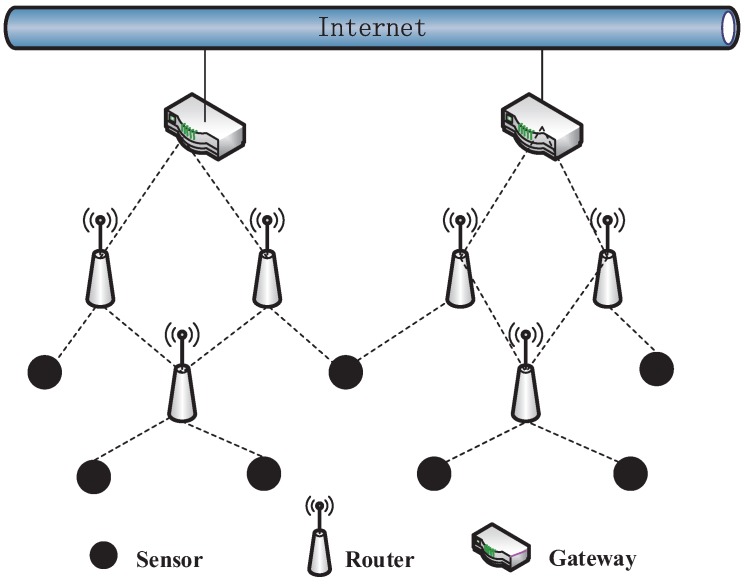
WMN gateway deployment using CPS.

CPS obtains the information of the physical world through many sensors and impacts the environment by actuators. Wireless networks have been widely used in cyber-physical systems (CPS) for data transmission. A CPS usually contains lots of sensors, and these sensors generate massive data. A number of special nodes must be utilized as the network gateway, collecting information acquired by the wireless sensory network and gaining access to the Internet, in order to achieve cross-region information acquisition, and WMN is a feasible wireless network architecture. Figure 1 shows a gateway deployment diagram that includes the entire network transmission model, consisting of sensor nodes, routing nodes and gateway nodes. The sensor nodes are used to sense environmental information, the routing nodes are used to forward information sent between nodes, and the gateway nodes are used to access the Internet. The network characteristic is that most of the traffic converges on the gateways. The service quality is better for nodes closer to the gateway node; however, for nodes far from the gateway node, the quality of service is poor. In other words, the quality of service is not equally distributed between nodes. Therefore, the gateway nodes often become the bottleneck of network performance, and whether the deployment of the gateway nodes is reasonable has a large impact on network performance.

A WMN is a self-organizing multi-hop mobile network in which the maximum communication coverage of an access point (AP) node is limited by the node’s transmission power. Thus, it is a circle with a fixed radius. AP nodes within the coverage radius are connected with one hop, forming a multi-hop network topology. The distance between a gateway and its farthest AP node has a great impact on network communication quality. The main goal of WMN gateway deployment is to maximize a network’s communication quality by adjusting the gateway location.

WMN topology can be represented by G(V,E), and all AP nodes in the network can be represented using a point set V(G). If the coordinates of a node $v_{i}$ are $\left(x_{i},y_{i}\right)$, in which $l_{ij}=\sqrt{{\left(x_{i}-x_{j}\right)}^{2}+{\left(y_{i}-y_{j}\right)}^{2}}$, then when the distance between any two nodes is no greater than *r*, namely, when $l_{ij}\leq r$, $v_{i}$ and $v_{j}$ can be considered as adjacent nodes. The connection between any two adjacent nodes in the network is an undirected edge whose weight is set to 1. Adjacent nodes are thus said to be connected by one hop. All inter-node edges constitute the edge set, E(G), of graph *G*.

A WMN must establish a gateway in order to provide service for all of the AP nodes. The service gateway for each AP node is then chosen based on which gateway is the closest i.e., is the fewest number of hops away. If it is assumed that all of the *k* gateway nodes have been selected in a network (*G*) of size *n*, *G*’s adjacency matrix is $A={\left\{e_{ij}\right\}}_{n\times n}$, where $e_{ij}\in E\left(G\right)$, and the shortest distance matrix is $D={\left\{d_{ij}\right\}}_{n\times n}$, in which $d_{ij}$ is the minimum number of hops from node $v_{i}$ to node $v_{j}$. If $d\left(v_{i},u_{j}\right)\leq d\left(v_{i},u_{l}\right),j,l\leq k,l\neq j$, then the AP node $v_{i}$ will choose gateway $u_{j}$ as its service gateway. Therefore, the service set of gateways, $u_{j}$, includes $v_{i}$, namely, $v_{i}\in U_{j}$. Th maximum distance between $u_{j}$ and the nodes in its service set $U_{j}$ is $\max_{v_{i}\in U_{j}}\left(d\left(u_{j},v_{i}\right)\right)$, which is referred to as the coverage radius of gateway $u_{j}$. The maximum coverage radius of a gateway node, $\max_{1\leq j\leq K}\max_{v_{i}\in U_{j}}\left(d\left(u_{j},v_{i}\right)\right)$, is called the coverage radius of the gateway set ${\left\{u_{i}\right\}}_{k}$, also known as the hop from the AP nodes to the gateway set.

The smaller the coverage radius of a gateway set is, the higher the quality of service the AP nodes will obtain. Therefore, the coverage radius of a gateway set is an important evaluation index for gateway deployment. The goal of WMN gateway deployment optimization is to minimize the coverage radius of a gateway set, that is, to minimize the path length. The model is as follows:

$$\begin{array}{cc} \min & \max_{1\leq j\leq K}\max_{v_{i}\in U_{K}}\left(d\left(u_{j},v_{i}\right)\right)\\ s.t. & {\left\{u_{j}\right\}}_{K}\subset R^{2}, \end{array}$$

where $R^{2}$ is the two-dimensional plane in which graph *G* is located.

## 3. Geometric *K*-Center Problem of WMN Gateway Deployment

There are two types of CPS WMN gateway deployment options: one is to choose the gateway from the *K* nodes in the existing network, which is referred to as the vertex *K*-center problem; the other option is to choose gateway nodes *K* positions from the plane in which the nodes are located, and these *K* gateway nodes are not necessarily existing network nodes, which is referred to as the geometric *K*-center problem. The former has a poor performance but is simple and easy to implement, while the latter performs better but is difficult to solve.

There are two types of CPS WMN gateway deployment: one is to choose *K* nodes from the existing network nodes to act as the gateways, called the vertex *K*-center problem. The other is to choose *K* positions from the plane in which the nodes are located to deploy gateway nodes, where these *K* gateway nodes are not necessarily existing network nodes, called the geometric *K*-center problem. The former is simple and easy to implement but results in poorer performance, while the latter performs better but is difficult to solve.

**Figure 2:**
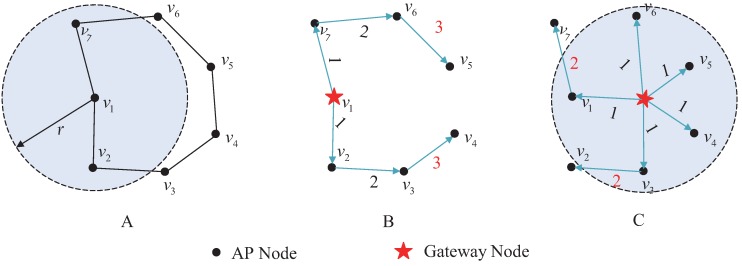
Gateway deployment of a 7-node network. (**A**) original network; (**B**) gateway deploying on node; (**C**) gateway deploying not on node.

Figure 2A is a 7-node network topology, in which the node degree is 2. The network forms a connected closed loop, and the node’s maximum coverage is a circle with a fixed radius. The weight of any given edge is set to be the number of hops between the gateway and the node. Figure 2B shows the gateway deployment method based on the vertex *K*-center problem, in which an AP node is selected from V(G) as a gateway, and the global optimal coverage radius is 3. Figure 2C shows the gateway deployment method based on the geometric *K*-center problem, in which a suitable location on the plane is chosen to place the gateway, and the coverage radius drops to 2.

**Figure 3:**
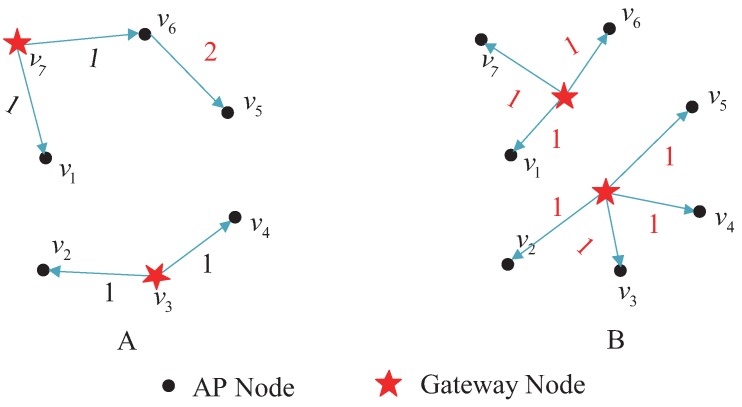
Deployment diagram of two gateways. (**A**) gateways deploying on nodes; (**B**) gateways deploying not on nodes.

The number of gateways deployed for the topology shown in Figure 2A is two. When the vertex *K*-center deployment approach is used, as shown in Figure 3A, the coverage radius for the network is two. When the geometric *K*-center deployment approach is used, as shown in Figure 3B, the gateway is deployed at the geometric center and the coverage radius drops to one.

According to the above analysis, the vertex *K*-center problem, which is simple and easy to implement, involves deploying each gateway at a WMN node; the geometric *K*-center problem involves deploying each gateway anywhere in the network plane, the flexibility in terms of gateway location can thus, theoretically, be used to achieve a global optimum.

## 4. CSO-Based Geometric *K*-Center Gateway Deployment in CPS

The PSO algorithm is a stochastic search and intelligence heuristic algorithm, which can search extremums in continuous high dimensional space, and has a balanced spatial and local search ability, which is fast and not easy to fall into the local optimal solution, so it is very suitable to use when solving the problem of WMN gateway deployment. However, because its search mechanism involves constant updates regarding the particles’ position and speed according to the individual optimum (pbest) as well as the population optimum (gbest), it is computationally expensive and is prone to falling into local optima and premature convergence. In order to address this issue, a competition mechanism can be used in order to update the particles instead of using the individual or population optimum [6]. Therefore, in this study, a competition-based CSO algorithm is adopted in order to solve the geometric *K*-center gateway deployment problem in a WMN.

### 4.1. Search Mechanism of the CSO Algorithm

**Figure 4:**
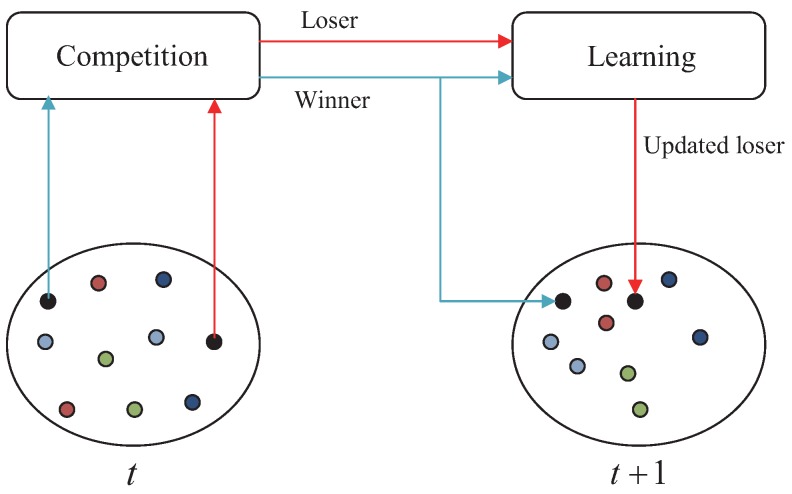
Search mechanism of the Competitive Swarm Optimizer (CSO) algorithm.

A swarm of *n* particles flying in an *m*-dimensional space and searching for an optimum can be randomly divided into $\frac{n}{2}$ pairs, assuming that *n* is an even number, at any point in time. Subsequently, competition exists between the two particles in the same pair and the particle that is fitter wins. The winner maintains its state and directly enters the next moment; the loser learns from the winner, updating both its position and speed, and then moves on to the next moment. In summary, at any point in time, each particle participates in the competition once, and $\frac{n}{2}$ particles will update their position and speed. The CSO algorithm, as compared to the PSO algorithm, reduces the computational cost. A schematic of the algorithm’s search mechanism is shown in Figure 4.

The pseudo code of the competitive swarm optimizer (CSO) algorithm (Alogorithm 1) is as follows:
**Algorithm 1** The Competitive Swarm Optimizer Algorithm
1:Initialize P(0),Maxgen; // P(t) is the swarm ingeneration *t*, Maxgen is the maximum number of iterations.2:**for** t=1 to Maxgen **do**3:  calculate the fitness of all particles in P(t);4:  L=P(t),P(t+1)=∅;//*L* denotes a set of particles that have not yet participated in a competition.5:  **while** L≠∅ **do**6:    randomly choose two particles $X_{1}\left(t\right),X_{2}\left(t\right)$7:    **if** $f\left(X_{1}\left(t\right)\right)\leq f\left(X_{2}\left(t\right)\right)$ **then**// $f(X_{1}\left(t\right))$ is the fitness function for $X_{1}\left(t\right)$8:     $X_{w}\left(t\right)=X_{1}\left(t\right),X_{l}\left(t\right)=X_{2}\left(t\right)$; // $X_{w}\left(t\right)$ is the winner particle in generation *t*, and $X_{l}\left(t\right)$ is the loser particle in generation *t*9:    **else**10:     $X_{w}\left(t\right)=X_{2}\left(t\right),X_{l}\left(t\right)=X_{1}\left(t\right);$11:    **end if**12:    add $X_{w}\left(t\right)$ into P(t+1);13:    update $X_{l}\left(t\right)$ using (1) and (2)14:    add the updated $X_{l}\left(t\right)$ into P(t+1);15:    remove $X_{1}\left(t\right),X_{2}\left(t\right)$ from *L*16:  **end while**17:**end for**


### 4.2. Encoding of the CSO Algorithm

The position of the *i*th particle in the CSO algorithm can be written as $X_{i}=\left(x_{i1},x_{i2},\ldots ,x_{im}\right)$. When solving a geometric *K*-center problem, the position of a particle in the CSO algorithm represents a set of solutions to the WMN gateway deployment problem, coded as the combination of the coordinates of *K* gateways’ in plane $R^{2}.$ The positions of the gateways’ are as follows: $\left(a_{1},b_{1}\right),\left(a_{2},b_{2}\right),\ldots ,\left(a_{j},b_{j}\right),\cdots ,\left(a_{K},b_{K}\right)$, in which $a_{j}=x_{2j-1},\;b_{j}=x_{2j}$ and m=2K. The flying speed of the *i*th particle is $S_{i}=\left(s_{i1},s_{i2},\cdots ,s_{im}\right)$.

### 4.3. Dynamic Equations of the CSO Algorithm

In each iteration of the CSO algorithm, the particles’ speeds and positions are updated to search for a better solution using a competition mechanism. The position of the winner of the *g*th pair at time *t* is $X_{w,g}\left(t\right)$, and its speed is $S_{w,g}\left(t\right)$, where $g=1,2,\ldots ,\frac{n}{2}$. The loser’s position and speed are $X_{l,g}\left(t\right)$ and $S_{l,g}\left(t\right)$, respectively. For the *g*th pair at time *t*, the position of the loser is updated according to Equation (1), and its speed is updated according to Equation (2), in which $r_{1}$, $r_{2}$, and $r_{3}$ are random numbers in ${\left[0,1\right]}^{m}$. $\bar{X}\left(t\right)$ is calculated as follows:

(1) $$\begin{array}{cc} & S_{l,g}\left(t+1\right)=r_{1}S_{l,g}\left(t\right)+r_{2}\left(X_{w,g}\left(t\right)-X_{l,g}\left(t\right)\right)+r_{3}\xi \left(\bar{X}\left(t\right)-X_{l,g}\left(t\right)\right), \end{array}$$

(2) $$\begin{array}{cc} & X_{l,g}\left(t+1\right)=X_{l,g}\left(t\right)+S_{l,g}\left(t+1\right). \end{array}$$

$\bar{X}\left(t\right)$ is the average position of the population at a given time, and *ξ* is used as a parameter to control $\bar{X}\left(t\right)$ and to increase the diversity of the population. The position of the particles that have lost the competitions adjusted towards the position of the particles that won, so that each particle reaches an optimal position. The particles that have won the competition maintain their positions and speed and directly enter the next stage.

### 4.4. Fitness Function Design in the CSO Algorithm

In the geometric *K*-center WMN gateway deployment problem, a decreased fitness value is indicative of better particles, and the fitness is determined by the coverage radius of the particle. A particle in the CSO algorithm is a combination of the position coordinates of *K* gateways. Considering that the particles can be placed in any position of the plane, calculating the distance from the particle to the AP node is difficult; the following method can be used.

The position of the *k*th gateway, $u_{k}$, of the particle is $\left(a_{k},b_{k}\right)$, where k=1,2,···,K. Nodes with a distance of $u_{k}$ less than the communication distance (*r*) form a set, referred to as the adjacent node set, $ADJ_{k}$. The distance from node $v_{i}$ to $u_{k}$ is $d\left(v_{i},u_{k}\right)=\min_{v_{j}\in ADJ_{k}}\left(d\left(v_{i},v_{j}\right)\right)+1$. The coverage radius of a particle, i.e., the fitness of the particle, is $F=\max_{1\leq i\leq n}\min_{1\leq k\leq K}\left(d\left(v_{i},ADJ_{k}\right)\right)+1$.

### 4.5. Solving the Gateway Location Problem Using the CSO Algorithm

In the CSO algorithm, particles are randomly generated at the beginning, with the coverage radius of the particle representing its fitness. The fitness of the particles is evaluated after each iteration, and the particles are then randomly paired for competition. The position of the particles that lose the competition is adjusted towards the position of the winning particles by changes made to their flying speeds and positions. Neither the position nor the speed changes for any of the winning particles and they directly enter the next iteration. When a fixed number of iterations have been completed, the algorithm will stop. The steps to solve the WMN gateway deployment problem using the CSO algorithm are as follows:

**Step 1**. A particle swarm of size *n*, including the positions $X_{i}$ and speeds $S_{i}$, is randomly generated within the effective area of the network graph in order to determine the value of *ξ*.
**Step 2**. Calculate the fitness of each particle, i.e., the coverage radius *R*.
**Step 3**. Randomly divide particles into $\frac{n}{2}$ groups. Two particles then compete against each other in each group, and the fitness of each particle is taken as the criterion for the outcome of the competition. The fitter particle is deemed the winner, while the other is the loser.
**Step 4**. Update the position and speed of the particles that lost the competition according to Equations (1) and (2). The winners retain their position and speed.
**Step 5**. Decide whether the termination condition of the algorithm is satisfied. If it is satisfied, stop the iteration and output the optimal solution. Otherwise, return to Step 2. The termination condition of the algorithm is to reach the maximum number of iterations.

## 5. Simulation Analyses

The platform used for the simulation in this section of the experiment was Matlab2012 running on a quad-core computer with 4 GB of memory and a 3.4 GHz CPU. Four random networks (50, 200, 600 and 1000 nodes) were used to simulate a WMN. All the random networks were connected, and the node degree of the networks was between 1 and 11. The experiment aimed to minimize the coverage radius using the CSO, PSO and Kmedoids algorithms at different network scales. Twenty independent experiments were conducted for each simulation, and the best, worst, mean and standard deviation of the coverage radius were recorded for each experiment. The stability of each algorithm was also analyzed.

### 5.1. AP Node Random Distribution

**Figure 5:**
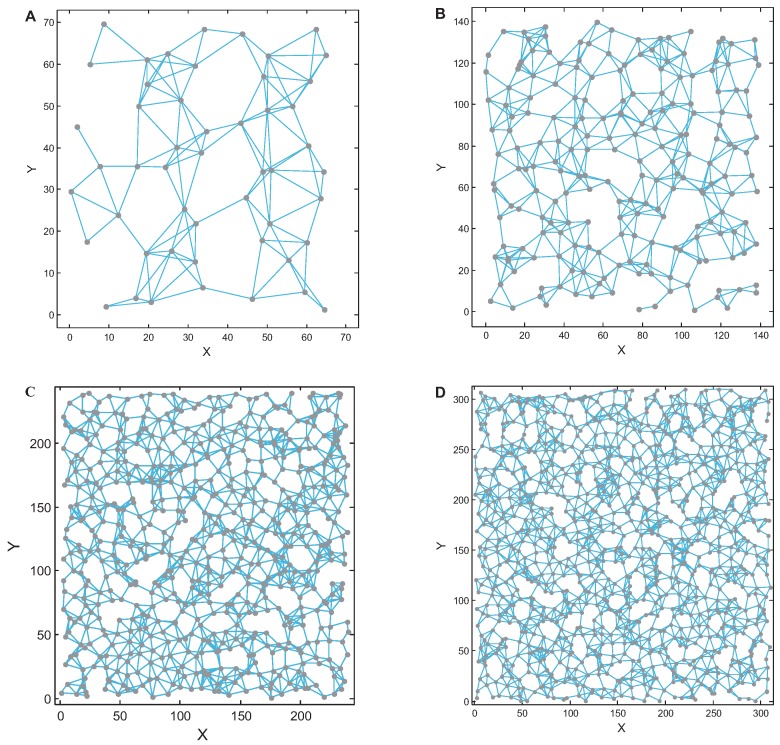
Gateway deployment of a 7-node network. (**A**) 50 nodes; (**B**) 200 nodes; (**C**) 600 nodes; (**D**) 1000 nodes.

Figure 5A–D shows diagrams of four random networks with 50, 200, 600 and 1000 nodes. The maximum degree of the graph is set to 11, and the minimum degree is 1. Each graph is connected. These four graphs span a large size range and are good data sets for testing the algorithms. In this paper, these four datasets were used to test the optimization performance of the CSO, PSO and Kmedoids algorithms.

### 5.2. Analysis of the Algorithms’ Optimization Performance and Convergence

The optimization results of the CSO algorithm, PSO algorithm and the Kmedoids algorithm for random WMNs comprised of 50, 200, 600 and 1000 nodes are shown in Table 1. The number of gateways was fixed at 5. The maximum, minimum, mean and standard deviation of the coverage radii in 20 independent experiments using the three heuristic algorithms are provided.

According to the results in Table 1, the CSO algorithm was shown to achieve a better maximum, minimum, and mean value when compared to the results obtained using the PSO algorithm and the Kmedoids algorithm. The standard deviation of the CSO algorithm was small, and the algorithm was stable. The CSO algorithm and the PSO algorithm both use similar mechanisms: they both find the optimal solution by particle searching in a continuous space, while the Kmedoids algorithm searches for the optimal solution among existing network nodes. The optimization performance of the Kmedoids algorithm was shown to be inferior to that of the CSO algorithm and PSO algorithm.

There are some differences in the search mechanism between the CSO algorithm and the PSO algorithm. The PSO algorithm updates the position and speed of the particles using the individual optimum (pbest) and the population optimum (gbest). Thus, all of the particles will be updated and moved towards a more optimal position. However, the population is prone to premature convergence. In contrast, when using the CSO algorithm, the individual optimum and the population optimum are discarded, and a competition mechanism is used instead: the losing particles learn only from the winning particles, effectively preventing local optimum trapping and, when compared to the PSO algorithm, this method effectively reduces the computation cost by only updating half of the particles in each iteration.

**Figure 6:**
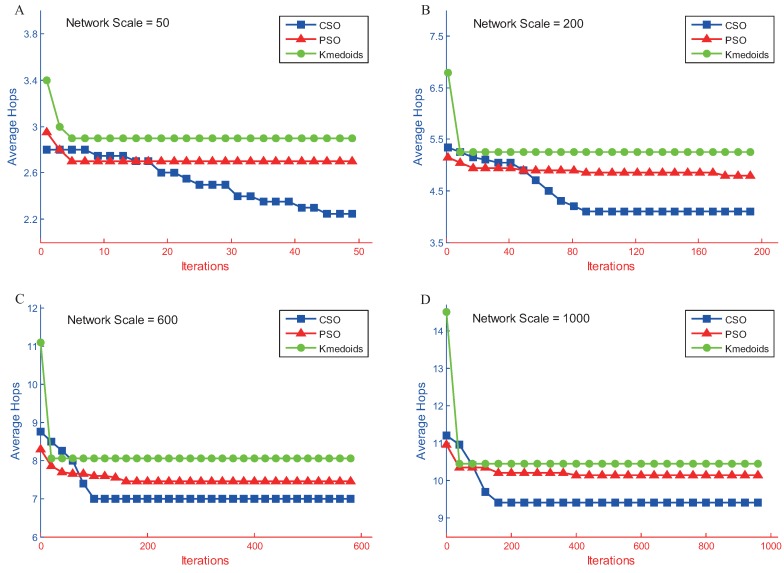
Convergence of three algorithms at different network scales. (**A**) 50 nodes; (**B**) 200 nodes; (**C**) 600 nodes; (**D**) 1000 nodes.

The average number of hops decreased as the number of iterations increased, as shown in Figure 6. After a certain number of iterations, the average number of hops tended to stabilize. When comparing the convergence of the three algorithms, the PSO algorithm and the Kmedoids algorithm were shown to converge prematurely and were prone to local optimum trapping. The CSO algorithm maintained the diversity of the population through the use of a competition mechanism, thus resulting in a longer convergence process that could effectively prevent local optimum trapping. Therefore, the CSO algorithm was shown to have a better performance in regards to solving the geometric *K*-center gateway deployment problem.

If the number of gateways is fixed, the gateway deployment performance graph does not show a significant improvement with an increasing network size. In the current study, networks comprised of 50 and 200 nodes were used to compare the performance of the algorithms. A total of five gateways were used. The first row in the figure represents the deployment performance of the three algorithms when the network size is 50 nodes; the second row represents the deployment performance when the network size is 200 nodes. The different colors in the figure represent different groups. The star symbol represents the gateway node, and the remaining symbols represent the AP nodes.

Figure 6 shows that the average number of hops decreased as the number of iterations increased; however, beyond a certain number of iterations, the average number of hops tended to stabilize. This comparison of the convergence of the three algorithms indicates that the PSO algorithm and the Kmedoids algorithm converged prematurely and were prone to local optimum trapping. The CSO algorithm ensured the diversity of the population through the competition mechanism. Consequently, its convergence process lasted longer and effectively prevented the population from becoming trapped in a local optimum. Therefore, the CSO algorithm showed better performance in solving the geometric *K*-center gateway deployment problem.

When the number of gateways is fixed, the gateway deployment performance graph does not show significant improvement as the network size increases. This paper used networks of 50 and 200 nodes to compare the algorithms’ performances with five gateways. The first row in Table 1 represents the deployment performance of the three algorithms with a 50-node network; the second row represents the deployment performance with a 200-node network. The different colors in the figure represent different groups. The star symbol represents gateway nodes, and the remaining symbols represent AP nodes.

**Figure 7:**
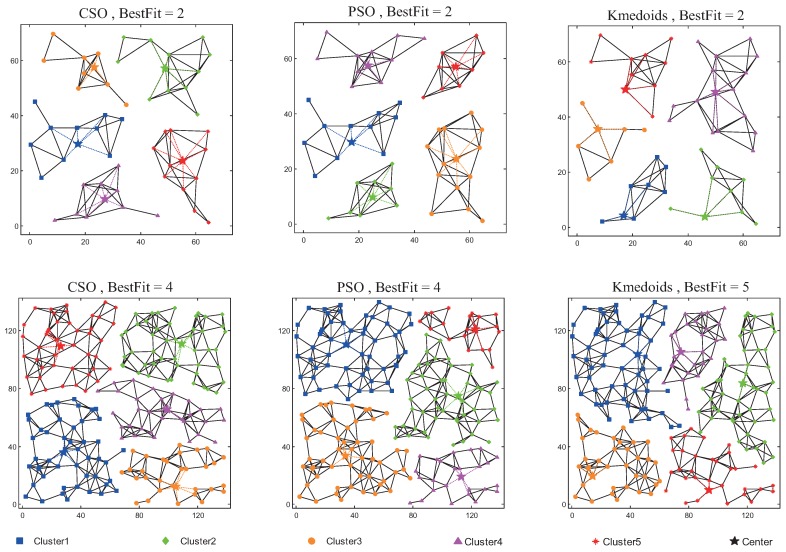
Optimization of three algorithms for networks of different sizes.

Figure 7 shows that the Kmedoids algorithm set up the gateways at client nodes while the CSO and PSO algorithms set the gateways at $R^{2}$, located in the geometric centers of the network diagram, and the number of AP nodes served by each gateway was relatively uniform, producing a relatively small coverage radius and balanced load among gateways.

### 5.3. Optimization Results of Algorithms with Different Numbers of Gateways

**Figure 8:**
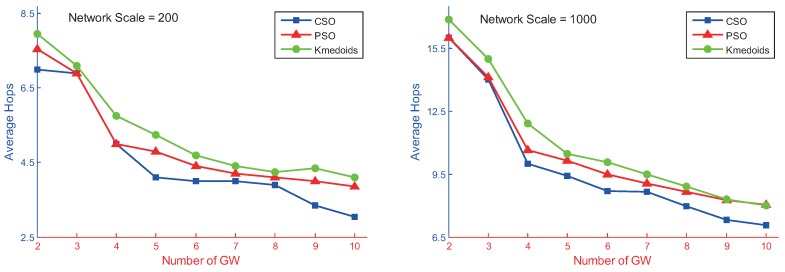
Number of hops of three types of algorithms at different network scales.

In this section, networks of 200 and 1000 nodes were taken as being representative of small- and large-scale networks. Figure 8 shows the performance of the CSO, PSO and Kmedoids algorithms when varying the number of gateways used for these two different random networks. Each experiment was independently run 20 times. The experimental results shown are the average of the 20 independent tests of each algorithm.

The above experimental results indicated that the coverage radius decreased as the number of gateways increased. For a fixed number of gateways, the coverage radius of the CSO algorithm was better than that of the PSO algorithm and Kmedoids algorithms. The CSO algorithm and PSO algorithm can be used to solve the geometric *K*-center gateway deployment problem, and their gateways are usually located at the center of multiple non-adjacent nodes, with a greater node degree. These two algorithms can shorten the routing distance between AP nodes and reduce the coverage radius of the network. The Kmedoids algorithm is suitable for solving the vertex *K*-center problem of WMN gateway deployment, where the gateway is selected from existing AP nodes. The analysis indicated that, from the perspective of coverage radius, the geometric *K*-center method performed better than the vertex *K*-center method in gateway deployment.

## 6. Conclusions

This study investigated the geometric *K*-center WMN gateway deployment problem in a CPS, or, more specifically, how to appropriately deploy gateways in a given geometric plane and reduce the routing distance from the AP nodes to the nearest gateway in order to improve the communication quality of the entire network. To address this problem, the target of optimization was the minimum coverage radius, which was solved using a new CSO algorithm. According to the experimental results, the CSO algorithm was shown to solve the geometric *K*-center problem of a WMN very well, providing a good solution to the geometric *K*-center gateway deployment.

## Figures and Tables

**Table 1:** Coverage radius of three algorithms at different node scales.

Node Scale	Algorithm	Best Value	Worst Value	Average Value	STDEV (Stand Deviation of Hop)
50	CSO	2	3	2.25	0.4443
	PSO	2	3	2.70	0.4702
	Kmedoids	2	4	2.90	0.5525
200	CSO	4	5	4.10	0.3078
	PSO	4	5	4.80	0.4104
	Kmedoids	5	7	5.25	0.6387
600	CSO	7	7	7	0
	PSO	7	8	7.45	0.5104
	Kmedoids	7	9	8.05	0.6863
1000	CSO	9	10	9.4	0.5026
	PSO	9	11	10.15	0.6708
	Kmedoids	10	15	10.45	1.1459
